# Coexistence of Cutaneous Squamous Cell Carcinoma and Basal Cell Carcinoma in a Renal Transplant Recipient: A Case Report

**DOI:** 10.7759/cureus.28764

**Published:** 2022-09-04

**Authors:** Diana Voloshyna, Tanveer Ahamad Shaik, Sunita Shrestha, Ajmat Ansari, Faraz Saleem, Muhammad Abu Zar Ghaffari

**Affiliations:** 1 School of Medicine, University of Michigan, Ann Arbor, USA; 2 Cardiovascular Medicine, University of Louisville School of Medicine, Louisville, USA; 3 Neurosurgery, Upendra Devkota Memorial National Institute of Neurological and Allied Sciences, Kathmandu, NPL; 4 Internal Medicine, Kathmandu University School of Medical Sciences, Dhulikhel, NPL; 5 Internal Medicine, Akhtar Saeed Medical and Dental College, Lahore, PAK

**Keywords:** renal transplant, post-transplant malignancy, concurrent non-melanoma skin cancers, ktr, scc, bcc, post-renal transplant, incidence and risk factors, basal cell carcinoma of scalp, squamous cell carcinoma

## Abstract

In solid organ transplant patients, non-melanoma skin cancer remains a leading cause of mortality. The most common skin malignancies in solid organ transplant patients are squamous cell carcinoma (SCC) and basal cell carcinoma (BCC). In organ transplant patients, SCC is 100 times more prevalent, and BCC is 10 times more prevalent than in the general population. Many risk factors for developing such malignancies are equivalent to those in the general population. However, in the transplant population, such cancers occur at an earlier age, act more aggressively, and often appear at multiple locations. Thus, assiduousness on the patient's part and healthcare providers is the highest priority. The concurrence of SCC and BCC together is rarely encountered in a post-transplant individual. We report a rare case of coexistence of SCC and BCC in the same patient. A 63-year-old man had been diagnosed with SCC and BCC simultaneously by a punch biopsy performed at two different scalp lesions of different diameters. This review describes an unusual occurrence of both skin cancers concurrently in a kidney transplant recipient.

## Introduction

Due to the rapid advancements in surgery and medicine, the number of kidney transplants is rising every year. However, lifelong immunosuppressive therapy after kidney transplantation may produce dermatological adverse effects. Skin cancer is the most common skin disease after organ transplantation [1].

There is a positive association between renal transplantation and an increased risk of non-melanoma skin cancer (NMSC) [2]. Interestingly, in patients who developed skin cancer after retransplantation, lesions were not only multiple but were also more aggressive and pronounced [3].

NMSC, particularly squamous cell carcinoma (SCC) and basal cell carcinoma (BCC), is one of the most common malignancies after kidney transplantation. Besides immunosuppressive use, other risk factors, including age, male gender, fair skin, ultraviolet (UV) exposure, and duration of immunosuppressants, are reported to be associated with the risk of developing NMSC in kidney transplant recipients (KTRs) [2]. Most transplant patients with a single diagnosed skin lesion have more than a 50% incidence of multiple new lesions in their lifetime [4]. In KTRs, the likelihood of subsequent skin cancers being the same as the first is much higher than in having two different types of NMSCs [5].

## Case presentation

**Figure 1:**
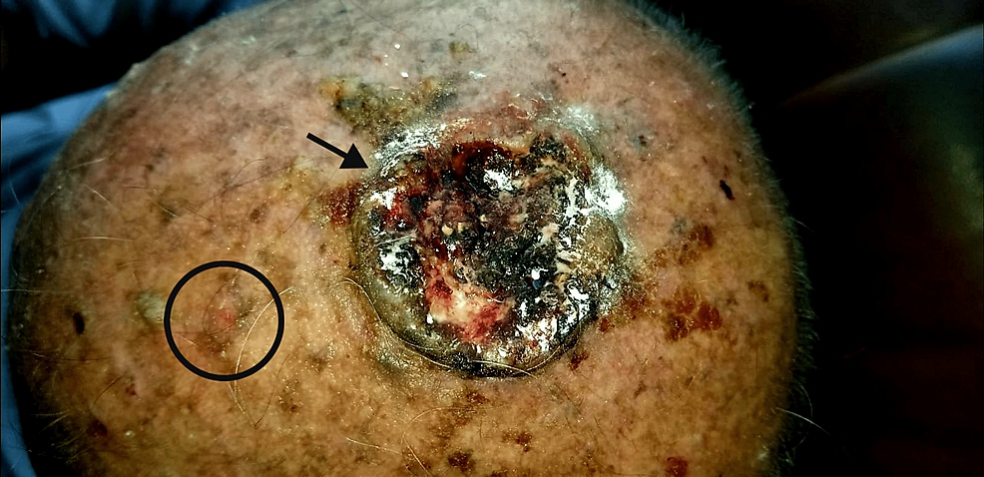
The arrow shows a large wound (squamous cell carcinoma) and the circle shows a small suspicious lesion (basal cell carcinoma)

A 63-year-old car mechanic presented with ulcerative growth on his scalp vertex. He had a history of a wound on the scalp from a local mechanical tool injury while working in his workshop eight months back. Over the course of seven months, the wound failed to heal and got worse despite applying self-prescribed local antibiotic powder and ointment. The patient subsequently noted an increase in the diameter of the wound, which led to worry, and he visited the local tertiary care hospital. Clinical examination revealed a large painless and ulcerated lesion measuring 3.2 x 2.8 cm in diameter along with a small suspicious superficial lesion (measuring 0.2 x 0.3 cm) 3 cm away from the larger lesion on the scalp. Variably sized pigmented macules and several seborrheic and actinic keratosis lesions were also seen on sun-exposed areas of the scalp. Figure 1 displays these findings.

Upon review of the patient's history, the patient had undergone a renal transplant 18 years ago and was on immunosuppressants and steroids for the last 15 years. He has been taking Imuran 50 mg and prednisolone 10 mg a day for the previous 15 years. Punch biopsy for both lesions was advised under the suspicion of SCC. Biopsy of the larger lesion on the scalp confirmed the growth to be a well-differentiated SCC. However, the biopsy of a smaller lesion on the scalp was suggestive of BCC.

**Figure 2:**
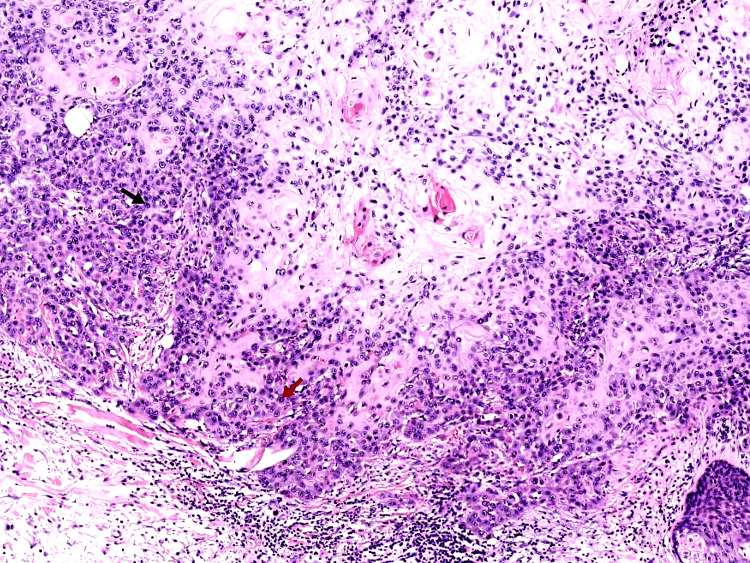
The black arrow shows keratinocytes and the red arrow shows enlarged nuclei in the eosinophilic background (hematoxylin & eosin, 100x)

**Figure 3:**
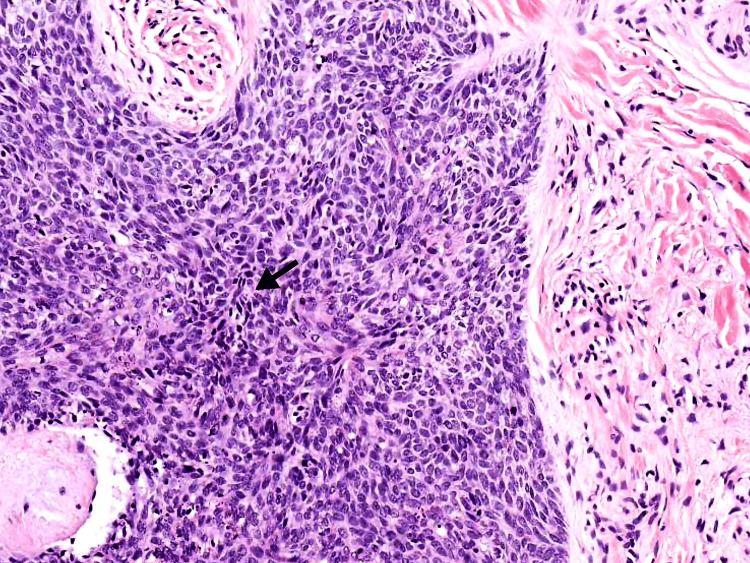
The black arrow shows a palisading pattern typical of basal cell carcinoma (hematoxylin & eosin, 200x)

The histopathology of the larger lesion showed islands and nests of polygonal tumor cells in the background of eosinophilic cytoplasm, keratinocytes, and hyper-chromatic nuclei with prominent nucleoli, suggestive of SCC. Figure 2 displays these findings. Biopsy of the smaller lesion revealed typical palisading growth where the outer cell layer of a tumor nest is made up of organized cells; the surrounding stroma is of a dense appearance characteristic of BCC. Figure 3 displays these findings. Based on the above presentation and his post-renal transplant history, a clinical diagnosis of SCC and BCC was made. The patient refused medical and oncological treatment and was referred to an appropriate surgical center for further management.

## Discussion

When two types of malignant tumors grow simultaneously in the same area, the combination of malignant melanoma and BCC is most commonly seen. The prevalence of coexisting tumors is very low, especially since the association between BCC and SCC has rarely been found [6]. It is estimated that SCC and BCC account for 20% and 80% of total cases of NMSC in the general population, respectively. In contrast to the general population, in KTRs, SCC is the most common, followed by BCC, with a more aggressive course and greater metastatic and recurrence potential [2,7]. A study observing 2075 Dutch recipients showed that 53% of recipients developed skin cancer [7].

The cumulative incidence of NMSC after transplantation increases over time [5]. In a study conducted in Australia, in patients more than 20 years post-transplant, the combined rate of SCC and BCC was 82% [8]. Subsequent skin cancer risk is relatively high in patients with existing NMSC. Compared to the general population, the risk was estimated to be 50 times higher in transplant patients within three and half years [9]. Interestingly a study in Britain stated that the mean interval for developing subsequent NMSC is 15 months after the appearance of the first skin cancer.

Moreover, this interval seems to decrease with the development of future skin cancers [5]. Previous studies focused mainly on the first SCC or BCC [5]. However, a study conducted on 222 KTRs with histological evidence of NMSC at a kidney transplantation center in the Netherlands found 86 recipients with SCC, 53 with BCC, and 83 who developed both. The incidence of developing subsequent skin cancers in KTRs was greater than 75%. In particular, 24 patients already had two to three NMSCs at the time of presentation to the clinic, and the frequency for the third and fourth NMSCs was greater than 50% [5]. In patients with prior skin cancer, the average duration of metastases was 1.4 years [9]. In one of the studies, significant fatalities were observed in patients with untreated metastases, and the survival rate was also reported to be 56% at three years [9]. Australian researchers also reported the aggressive cancerous disease. In one study, Veness et al. reported that 41% of the recipients developed skin cancers, and more than 50% of these patients died. In another Australian study, the cause of death due to skin cancer is 27% in KTRs [10].

Potential risk factors that are reported to be associated with the risk of NMSC in KTRs include male gender, smoking, advancing age, sun exposure, severe sunburns, genetic factors, increased UV light sensitivity, subtropical region inhabitants, and human papillomavirus (HPV) infection transmission before transplantation [2,7,9]. SCC usually presents as a single lesion; however, some patients may also have multiple lesions along with actinic keratosis [4]. Age played a pivotal role in the presentation of the site of lesions. In patients before the age of 40, the lesions were predominantly on the hands, upper limbs, and trunk dorsum. However, after this age, most skin cancers present in the head and neck area [9]. Clinical manifestations are infiltrative, ulcerative, or hemorrhagic nodular lesions, mainly on sun-exposed sites [9]. Male gender has been documented as a more significant risk factor for multiple NMSCs [5]. The higher chance of NMSC in the male sex is associated with higher levels of sun exposure [7]. The duration of immunosuppressant agents and advanced age at transplant are related to higher risks [9]. Other risk factors include the site of the lesion, especially the head/neck, tumor size, the multiplicity of lesions, specific histology, and the presence of tumors in extracutaneous regions [3]. Sunlight exposure is strongly associated with BCC [5]. Field cancerization is of critical significance in the development of NMSC. One example of field cancerization is actinic keratosis, which can present with numerous such lesions in a single anatomic area [4].

One of the significant risk factors in transplant patients is an impaired immune system, which predisposes the patient to develop NMSC primarily due to compromised immune surveillance [9]. The risk was almost 10 times greater in patients using immune-suppressive for 10 years or more than in those with five years or less [8]. Compared to the other immunosuppressive drugs, azathioprine and calcineurin inhibitors are associated with a greater incidence of skin cancers in KTR [9]. A decline in immunosurveillance is primarily associated with increased skin cancers. Recent studies have proven that modifications in immunosuppression should be repeatedly introduced in KTRs to lessen the risk of subsequent skin cancers [3].

## Conclusions

The inevitable use of newer and more potent immunosuppressive drugs and enhanced survival rates signify that the incidence of NMSCs will continue to rise in KTRs. The substantial burden of NSMCs in KTRs draws attention to the importance of early prevention and timely intervention of these skin lesions, which can confer significant morbidity and mortality benefits to this population. The ability to precisely identify individuals at risk of developing subsequent lesions after the initial skin diagnosis could be a valuable acquisition for targeted surveillance. Likewise, educating the patient about skin concerns, stressing the need for intensive sun protection, detailed physical examination of exposed areas, immunosurveillance monitoring, and regular follow-ups are of the utmost importance in the transplant population.
